# Comparison of Transoral Robotic Thyroidectomy and Transoral Endoscopic Thyroidectomy via Vestibular Approach Using an Endoscopic Retractor: A Single-Center Experience

**DOI:** 10.3390/cancers18020238

**Published:** 2026-01-13

**Authors:** Jun Sung Lee, Mun Chae Choi, Nam Kyung Kim, Hyeok Jun Yun, Seok-Mo Kim, Yong Sang Lee, Hang-Seok Chang

**Affiliations:** 1 Department of Surgery, Thyroid Cancer Center, Gangnam Severance Hospital, Institute of Refractory Thyroid Cancer, Yonsei University College of Medicine, Seoul 06263, Republic of Korea; ljs-513@yuhs.ac (J.S.L.);; 2 Department of Surgery, The Research Institute for Transplantation, Yonsei University College of Medicine, Seoul 03722, Republic of Korea

**Keywords:** TORT, TOETVA, thyroid cancer, endoscopic surgery, robotic surgery

## Abstract

**Background/Objectives**: The TOETVA and TORT have been successfully refined in recent years, with few complications and excellent results. In this study, we compared TORT and TOETVA in patients with papillary thyroid cancer. **Methods**: From September 2016 to January 2022, we retrospectively compared patient characteristics and clinical features from 100 TORT cases and 300 TOETVA cases, as well as their respective learning curves. **Results**: Median operation time of TORT was 80.4 min, similar to TOETVA (81.4 min, *p* = 0.719). Median docking time of TORT was 4.51 min and median console time was 27.72 min. The length of stay of TORT and TOETVA was 2.29 and 2.54 (*p* = 0.002). The median number of retrieved central lymph nodes for TOETVA and TORT were 2.89 and 2.99 (*p* = 0.746). **Discussion**: There was no significant difference with clinical features between TORT and TOETVA.

## Simple Summary

This study compares two scar-free thyroid cancer surgeries performed through the mouth—one using an endoscope and the other using a robotic system. All surgeries were performed by a single experienced surgeon using a specialized retractor. Both methods were found to be safe and effective, with similar surgical outcomes. While robotic surgery offers better precision, it is significantly more expensive. Therefore, the endoscopic approach can be a strong alternative for patients seeking safe, cost-effective treatment. These findings may help guide future choices in thyroid cancer surgery and expand options for patients.

## 1. Introduction

A variety of minimally invasive techniques for thyroidectomy have been introduced with the aim of preventing visible cervical scarring, most notably endoscopic and robotic procedures [1,2,3,4]. Among various remote-access thyroidectomy techniques, the transoral endoscopic thyroidectomy vestibular approach (TOETVA) has undergone continuous advancements in recent years, leading to enhanced surgical safety and efficacy. Initial clinical applications demonstrated that TOETVA offers excellent cosmetic outcomes with minimal invasiveness and a low incidence of complications [5]. Subsequent prospective studies confirmed its oncological safety in patients with differentiated thyroid cancer, with favorable postoperative recovery and functional outcomes [6]. Moreover, the introduction of three-dimensional imaging guidance for optimizing port placement has improved the precision of instrument handling and contributed to reducing the risk of nerve injury [7]. Comparative studies between vestibular and submental approaches have also shown that the vestibular route provides superior aesthetic results without compromising surgical effectiveness [8]. Collectively, these findings support TOETVA as a safe, effective, and cosmetically favorable minimally invasive surgical option.

The growing interest in TOETVA can be attributed to several advantages [9,10]. Firstly, it yields better cosmetic results than conventional techniques and other endoscopic procedures because the incisions are placed within the oral mucosa [11]. Secondly, flap dissection is more limited than with other remote-access approaches. Moreover, it utilizes a midline approach, thereby providing easier access to both thyroid lobes, unlike transaxillary and retroauricular techniques, which employ a lateral approach [10]. Our previous study showed that TOETVA with an endoscopic retractor could be performed safely and radically in selected patients with thyroid cancer [12].

In contrast, transoral robotic thyroidectomy (TORT) via a vestibular approach has become an important option of procedure over the last decade because robotic systems offer magnified three-dimensional imaging, making it easy to identify the parathyroid glands and the recurrent laryngeal nerve (RLN) and leading to safer, more precise dissection [13]. In 2020, we started performing TORT at our hospital and developed our robotic technique using an endoscopic retractor similar to that described in our previous report on TOETVA [12]. Recent meta-analyses have further highlighted the comparable efficacy and safety of TOETVA and TORT in selected patients [14,15]

To the best of our knowledge, large-scale analyses of more than 300 TOETVA cases and 100 TORT cases in thyroid cancer surgery by a single surgeon have not been reported [16,17]. In this study, we compared TORT and TOETVA conducted with a surgical method using three trocars and an endoscopic retractor in patients with papillary thyroid cancer (PTC).

## 2. Materials and Methods

### 2.1. Enrolled Patients

The medical records of 403 patients who underwent TOETVA or TORT for PTC between September 2016 and January 2022 at the Thyroid Cancer Center, Department of Surgery, Gangnam Severance Hospital, Yonsei University College of Medicine were retrospectively reviewed. All operations were performed by a single surgeon (S-M Kim).

The first TORT procedure was performed on 10 March 2020, at which point 217 TOETVA procedures had already been performed since 12 September 2016. At that time, the surgeon had become skilled and proficient and had overcome the learning curve by completing 217 TOETVA cases.

**Figure 1:**
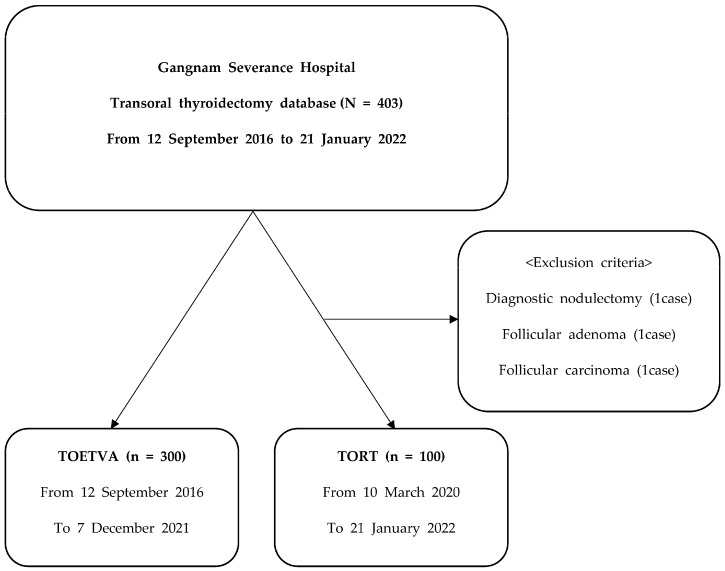
Flow diagram of patients.

Patients diagnosed with PTC who required hemithyroidectomy, isthmusectomy, or total thyroidectomy were included. Patients diagnosed with FTC and follicular adenoma who required diagnostic nodulectomy were excluded. After excluding 1 case of diagnostic nodulectomy, 1 case of follicular adenoma, and 1 case of follicular carcinoma following pathology, 100 TORT procedures were performed from 10 March 2020 to 21 January 2022. The 100 consecutive TORT cases were compared with 300 TOETVA cases (Figure 1).

Patients presenting with suspected capsular invasion or lymph node metastasis on preoperative imaging studies, including neck ultrasonography (US) and computed tomography (CT), were considered unsuitable candidates for the transoral approach and were therefore excluded from the present analysis.

This study was reviewed and approved by the Institutional Review Board of Gangnam Severance Hospital, Yonsei University College of Medicine (Seoul, Republic of Korea). Owing to the retrospective design of the study, the requirement for informed consent was waived.

### 2.2. Surgical Procedures

**Figure 2:**
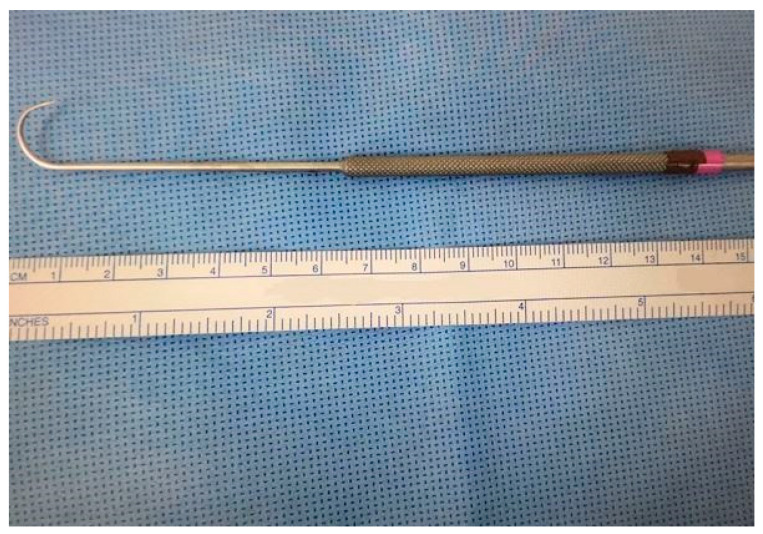
Endoscopic retractor developed for retraction of strap muscle.

**Figure 3:**
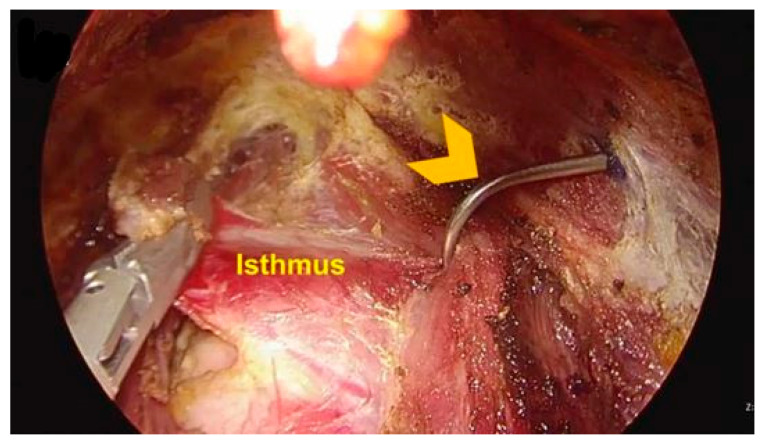
Placing an endoscopic retractor around the strap muscle (yellow arrowhead).

In this study, all cases were performed using an endoscopic thyroid retractor (Figure 2). A retractor device was developed for retraction of strap muscle for transoral thyroidectomy (Patent KR Registration 30-0926522). The endoscopic retractor was used to freely shift between the superior and inferior layers of strap muscle as required during surgery. By using an endoscopic retractor, surgeons can easily obtain the view needed for surgery without the use of unnecessary additional ports or other equipment. The endoscopic retractor was placed around the strap muscle during the surgery (Figure 3).

Until the robot docked, preoperative preparation for TORT was the same as that for TOETVA, as previously reported [8]. In TORT cases, a da Vinci Xi robot camera was inserted instead of the laparoscopic camera, and before docking the da Vinci Xi system, laparoscopic instruments were used for flap dissection as with TOETVA. The three trocars were changed from laparoscopic instruments to robotic instruments, and the da Vinci Xi system was docked using three robot arms. The camera was docked in the middle arm, the ultrasonic energy device (harmonic scalpel) was docked in one lateral arm, and the grasping device (prograsper), which could use bipolar energy, was docked in the other lateral arm. After the da Vinci Xi robotic system was undocked, the resected specimen was placed into an endoplastic bag as with TOETVA. After undocking, the surgical procedure of TORT was the same as that of TOETVA. A drain was not inserted for thyroid lobectomy via TOETVA and TORT. A simple dressing was applied to the skin aperture to insert the endoscopic retractor.

### 2.3. Outcome Measurement

Patient characteristics, pathologic findings, and clinical outcomes, including total operative time, were obtained by the analysis of medical records. Postoperative assessment of mental nerve function was conducted both during hospitalization and at outpatient follow-up visits. Patients were specifically questioned regarding the presence of sensory disturbances, such as numbness in the chin or lower lip region. Recurrent laryngeal nerve (RLN) integrity was evaluated using laryngoscopic examination, with vocal cord mobility serving as an indicator of nerve injury.

### 2.4. Statistical Analysis

Categorical variables were analyzed using Fisher’s exact test, while continuous variables were compared using an independent two-tailed t-test. A *p*-value of less than 0.05 was defined as statistically significant. To evaluate the learning curve, the slope derived from linear regression analysis of operative time across cases 1 to 10 (representing the skill acquisition phase) was compared with that of the subsequent 90 cases (proficiency phase) in the operative time–case number model. All statistical analyses were performed using SPSS software (version 22.0; IBM Corp., Armonk, NY, USA). Continuous data are presented as mean ± standard deviation, whereas categorical data are expressed as frequencies and percentages.

## 3. Results

### 3.1. Baseline Characteristics

A total of 400 cases of TOETVA and TORT were performed by a single surgeon. Baseline characteristics of the patients are summarized in Table 1. Of the 400 patients, 370 (92.5%) were female. The median age was 35.6 years. The mean body mass index (BMI) was 22.7 ± 3.550. According to the American Society of Anesthesiologists (ASA) classification system, 56.7% of patients belonged to class III. Total thyroidectomy was performed in 25 patients (6.3%), and less than total thyroidectomy was performed in 372 (93.0%) patients (326 underwent hemithyroidectomy, 46 underwent isthmusectomy). The median tumor size was 0.71 cm, and the mean number of retrieved parathyroid glands was 0.28 ± 0.479. The mean number of retrieved central lymph nodes was 2.91 ± 2.761, and the mean number of positive central neck lymph nodes was 0.49 ± 1.142. The median length of stay (days) was 2.48, and the median operation time (min) was 81.22. Final pathology analysis identified 393 (98.3%) patients with PTC. In terms of histological subtype, 374 (93.5%) cases were conventional. In addition, there were 16 cases of follicular variant, 3 cases of tall cell variant, 2 cases of encapsulated variant, 1 case of diffuse sclerosing variant and 1 case of solid variant.

### 3.2. Comparison Between TOETVA and TORT

The preoperative and intraoperative variables of TORT and TOETVA are compared in Table 2. Of the 400 patients, 100 underwent TORT and 300 underwent TOETVA. Sex, age, BMI, and extent of surgery did not differ between the two groups. The mean tumor size was greater in the TORT group than in the TOETVA group (0.83 ± 0.524 cm vs. 0.67 ± 0.345 cm, respectively, *p* = 0.004). The median operation time of TORT was 80.4 min, which was similar to that of TOETVA (81.4 min, *p* = 0.719). The median docking time of TORT was only 4.51 min, and the median console time was 27.72 min. The length of stay of TORT and TOETVA was 2.29 and 2.54, respectively (*p* = 0.002). The mean number of retrieved parathyroid glands was not significantly different between the two groups (0.23 ± 0.468 vs. 0.30 ± 0.482, *p* = 0.180). The incidence of lymphocytic thyroiditis was higher in the TORT group.

The comparisons of the number of retrieved central lymph nodes and positive central lymph nodes between TORT and TOETVA are presented in Table 3. The median numbers of retrieved central lymph nodes of TORT and TOETVA were 2.99 ± 2.827 and 2.89 ± 2.843, respectively, which showed no significant difference (*p* = 0.746). The median number of positive central neck lymph nodes of TORT was 0.78 ± 1.721, which was relatively larger than that of TOETVA (0.40 ± 0.850, *p* = 0.034).

**Figure 4:**
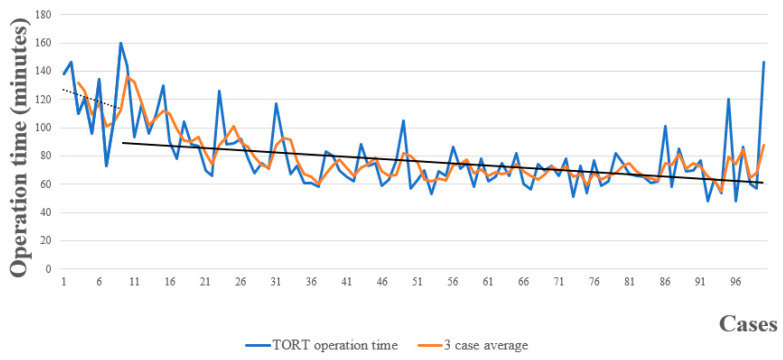
Learning curve in TORT operation time (min) in 100 consecutive cases. The slope of the skill acquisition period (cases 1–10) is depicted by the dashed black line, while the slope of the proficiency period (cases 11–100) is denoted by the solid black line.

The operation times for individual patients are plotted in chronological sequence in Figure 4. A simple moving average over 3 patients was calculated for 100 consecutive TORT cases. The slope of the skill acquisition period (cases 1–10) is depicted by the dashed black line, while the slope of the proficiency period (cases 11–100) is denoted by the solid black line.

## 4. Discussion

TORT via a vestibular approach has become an important option of procedure over the last decade. Unlike endoscopic thyroidectomy, robotic thyroidectomy provides fine motion scaling, hand-tremor filtering, innovative instrumentation with extended freedom of motion, and surgical education [13].

There are many prerequisites for performing robotic surgery. For example, it is possible only in hospitals equipped with robotic instruments. In addition, since robotic surgery is not covered by government health insurance in South Korea, patients have to pay three times the operating cost of TOETVA. Given the economic inaccessibility of TORT, TOETVA by highly skilled surgeons is considered a good alternative for patients who cannot afford it.

Overall, there were no significant differences between the TORT and TOETVA results in this study. There were no statistically significant differences in the number of retrieved central lymph nodes or the number of retrieved parathyroid glands between the two methods. The average length of stays (days) was shorter in TORT than TOETVA. (2.29 ± 0.656 vs. 2.54 ± 0.798, *p* = 0.002). It can be thought to indicate a slightly faster recovery of patients who underwent TORT compared to those who underwent TOETVA. However, given that the absolute difference in hospital stay is only approximately 0.25 days (2.29 vs. 2.54 days), it should be interpreted with caution, and further studies are needed to determine whether this minor variation translates into meaningful clinical or patient-centered benefits. There were several statistically significant differences between the two groups. The TORT group had larger tumors (*p* = 0.004), a higher proportion of male patients (*p* = 0.021), and a lower incidence of lymphocytic thyroiditis (*p* = 0.002) compared to the TOETVA group. While these differences are not expected to have substantially influenced the surgical procedures themselves, they may act as potential confounding factors when interpreting postoperative outcomes. Therefore, these baseline differences should be considered when assessing the comparative results between the two approaches.

Most previous studies included several benign cases (thyroid goiter, Grave’s disease, benign nodules), but this study was conducted in patients with thyroid cancer (PTC) only [18,19,20,21]. Additionally, unlike previous studies, this study was conducted with a surgical method using three trocars and an endoscopic retractor, and this is the first study to compare TORT and TOETVA using this surgical method.

The operation time of TORT was similar to that of TOETVA. In TORT, docking times were between 3 and 7 min, with a median docking time of only 4.51 min. The median console time (after strap muscle division to specimen removal) was 27.72 min. The operation time, docking time, and console time of TORT were shorter than those reported for other remote-access surgical approaches to the thyroid. In robotic thyroid surgery using a gasless, transaxillary approach, the mean operation time was 144 [69–347] min, the mean docking time was 6.4 ± 4.6 min, and the mean console time was 59.1 ± 25.7 min [4]. Meanwhile, in robot thyroidectomy by bilateral axillo-breast approach, the mean console time was 75 ± 26 min [22], the mean docking time was 16.5 ± 10.1 min, and the mean operation time was 184.9 ± 41.8 min [23].

We considered the first 10 cases of the series as a skill acquisition period for TORT, as we did for TOETVA in our previous study [8]. This is consistent with the estimated learning curve of 7 to 11 cases reported anecdotally for other surgeons learning the TOETVA procedure [5,24].

The surgeon who performed all surgeries in our study had substantial experience with 217 cases of TOETVA, as well as experience performing conventional thyroid surgery. Like that of TOETVA, the observed learning curve for TORT in the present study was substantially shorter than that described in previous studies [10,25]. In addition, the learning curve for TORT was shorter than that described for other remote-access surgical approaches to the thyroid (transaxillary, bilateral axillo-breast, and retroauricular) [4,26,27,28,29,30]. This suggests that a surgeon with considerable experience with TOETVA can easily perform TORT. However, for surgeons unfamiliar with TOETVA, the learning curve for TORT will be much steeper than that observed in this study.

In our study, TORT and TOETVA have similar features in preoperative and intraoperative variables. However, TORT has an excellent advantage in overcoming the limitation of the operator such as hand tremor due to the weight of the endoscopic instruments and identifying surgical anatomy by three-dimensional imaging, and it helps the operator to perform more sophisticated operations. Although there seems to be no significant difference between TORT and TOETVA now, it is thought that a further study will be needed to see the results if cases increase and data on surgical outcome are collected in the future.

Our study had several limitations. First, it was a retrospective study conducted at a single institution by a single surgeon with considerable experience in both TOETVA and conventional thyroidectomy, which may limit the generalizability of the findings to other settings or surgeons with different levels of experience. Second, long-term oncological outcomes, such as recurrence rate and disease-free survival, were not evaluated due to the relatively short follow-up period. Future studies with extended follow-up are warranted to validate the long-term oncological outcomes, including recurrence patterns and survival after TORT. Such follow-up studies are currently underway at our institution to collect long-term surveillance data and further evaluate the oncological safety of TORT. Third, there was a potential temporal bias due to the different time periods during which the two surgical techniques were introduced. TOETVA cases were initiated in 2016, while TORT began in 2020. Over time, the surgeon’s skills and operative techniques may have improved, which could have influenced the outcomes in favor of TORT. Although all procedures were performed by the same surgeon, the learning curve effect and procedural refinements over the years could not be completely controlled.

## 5. Conclusions

In this large single-center study, we found that TORT with an endoscopic retractor can be performed safely and radically in selected patients with thyroid cancer. We also found that TORT did not differ from TOETVA with an endoscopic retractor in terms of postoperative complication and operation time. Given the high cost of TORT, TOETVA by highly skilled surgeons can be considered a good alternative for patients who cannot afford it.

## Figures and Tables

**Table 1:** Baseline characteristics of patients.

Variable	*n* = 400
Sex (female)	370 (92.5%)
Age, years	35.6 ± 7.812
Body mass index, kg/m^2^	22.7 ± 3.550
ASA classification	
I	164 (41.0%)
II	227 (56.7%)
III	9 (2.3%)
**Extent of surgery**	
Total thyroidectomy	25 (6.3%)
Hemithyroidectomy	326 (81.5%)
Isthmusectomy	46 (11.5%)
Tumor size, cm	0.71 ± 0.403
Number of retrieved parathyroid glands	0.28 ± 0.479
Number of retrieved lymph nodes	2.91 ± 2.761
Positive central neck lymph nodes	0.49 ± 1.142
Length of stay, days	2.48 ± 0.772
Operation time, min	81.22 ± 26.243
**Final pathology**	
Papillary thyroid cancer (histological subtype)	393 (98.3%)
Conventional	374 (93.5%)
Follicular variant	16 (4.0%)
Tall cell variant	3 (0.8%)
Encapsulated variant	2 (0.5%)
Diffuse sclerosing variant	1 (0.3%)
Solid variant	1 (0.3%)
Lymphocytic thyroiditis	121 (30.2%)

The data are presented as n (%) or mean ± standard deviation.

**Table 2:** Comparison of preoperative and intraoperative variables between TORT and TOETVA. The data are presented as n (%) or mean ± standard deviation.

Variable	TORT (*n* = 100)	TOETVA (*n* = 300)	*p*-Value
Sex (female)	86 (86.0%)	284 (94.7%)	**0.021**
Age, years	34.3 ± 7.612	36.0 ± 7.845	0.061
Body mass index, kg/m^2^	23.1 ± 3.792	22.5 ± 3.460	0.152
Extent of surgery			
Total thyroidectomy	7 (7.0%)	18 (6.0%)	
Hemithyroidectomy	77 (77.0%)	249 (83.0%)	
Isthmusectomy	13 (13.0%)	33 (11.0%)	
Tumor size, cm	0.83 ± 0.524	0.67 ± 0.345	**0.004**
Length of stay, days	2.29 ± 0.656	2.54 ± 0.798	**0.002**
Operation time, min	80.4 ± 24.302	81.4 ± 26.891	0.719
Docking time, min	4.51 ± 2.680		
Console time, min	27.72 ± 19.586		
Number of retrieved parathyroid glands	0.23 ± 0.468	0.30 ± 0.482	0.180
Lymphocytic thyroiditis	19 (19.0%)	102 (34.0%)	**0.002**

Significant *p*-values (*p* < 0.05) are shown in bold text.

**Table 3:** Comparison of retrieved central lymph nodes and positive central lymph nodes between TORT and TOETVA.

Variable	TORT (*n* = 100)	TOETVA (*n* = 300)	*p*-Value
Number of retrieved central lymph nodes	2.99 ± 2.827	2.89 ± 2.843	0.746
Positive central lymph nodes	0.78 ± 1.721	0.40 ± 0.850	**0.034**

The data are presented as mean ± standard deviation. Significant *p*-values (*p* < 0.05) are shown in bold text.

## Data Availability

Some or all datasets generated during and/or analyzed during the current study are not publicly available but are available from the corresponding author on reasonable request.

## Abbreviations

The following abbreviations are used in this manuscript:

ASA	American Society of Anesthesiologists
CT	Computed tomography
IQR	Interquartile range
FTC	Follicular thyroid cancer
PTC	Papillary thyroid cancer
RLN	Recurrent laryngeal nerve
TOETVA	Transoral endoscopic thyroidectomy vestibular approach
TORT	Transoral robotic thyroidectomy
US	Ultrasound
